# Cytokine-Mediated Inflammation in the Oral Cavity and Its Effect on Lipid Nanocarriers

**DOI:** 10.3390/nano11051330

**Published:** 2021-05-18

**Authors:** Carolin Tetyczka, Sonja Hartl, Ramona Jeitler, Markus Absenger-Novak, Claudia Meindl, Eleonore Fröhlich, Sabrina Riedl, Dagmar Zweytick, Eva Roblegg

**Affiliations:** 1 Institute of Pharmaceutical Sciences, Department of Pharmaceutical Technology and Biopharmacy, University of Graz, 8010 Graz, Austria; carolin.tetyczka@uni-graz.at (C.T.); sonja.hartl@uni-graz.at (S.H.); ramona.jeitler@uni-graz.at (R.J.); 2 Center for Medical Research, Medical University of Graz, 8010 Graz, Austria; markus.absenger@medunigraz.at (M.A.-N.); claudia.meindl@medunigraz.at (C.M.); eleonore.froehlich@medunigraz.at (E.F.); 3 Institute of Molecular Biosciences, Biophysics Division, University of Graz, 8010 Graz, Austria; sabrina.riedl@uni-graz.at (S.R.); dagmar.zweytick@uni-graz.at (D.Z.)

**Keywords:** TR146, nanostructured lipid carriers, in-vitro inflammation, cytokine, lining mucosa

## Abstract

Topical drug administration to the oral mucosa proves to be a promising treatment alternative for inflammatory diseases. However, disease-related changes in the cell barrier must be considered when developing such delivery systems. This study aimed at investigating the changes in the lining mucosa caused by inflammation and evaluating the consequences on drug delivery systems such as nanostructured lipid carriers (NLC). For this, TR146 cells were treated with inflammatory cytokines and bacterial components. Cell viability and integrity, reactive oxygen species (ROS), and interleukin (IL)-8 release were used as endpoints to assess inflammation. Translocation of phosphatidylserine, cytoskeletal arrangement, opening of desmosomes, and cell proliferation were examined. Transport studies with NLC were performed considering active and passive pathways. The results showed that IL-1ß and tumor necrosis factor α induced inflammation by increasing IL-8 and ROS production (22-fold and 2-fold). Morphologically, loss of cell–cell connections and formation of stress fibers and hyperplasia were observed. The charge of the cell membrane shifted from neutral to negative, which increased the absorption of NLC due to the repulsive interactions between the hydrophobic negative particles and the cell membrane on the one hand, and interactions with lipophilic membrane proteins such as caveolin on the other.

## 1. Introduction

Oral health is an essential indicator of general health, well-being, and quality of life. Mechanical stress and chemical toxicants might change the homeostasis in the mouth leading to a number of conditions such as tooth decay, periodontal disease, stomatitis, cancer, oral mucositis, infections, xerostomia, and others. It is estimated that oral diseases affect 3.5 billion people worldwide and are among the fourth most common diseases in Europe, with annual costs of €79 billion [1,2].

A hallmark of oral diseases is inflammation, which occurs as a physiological reaction of the human body to pathogenic organisms, injuries, antigens, and ionizing radiation [3]. It can be either of short duration, and hence acute, which is characterized by fluid exudation and emigration of leukocytes, or prolonged and chronic. Chronic inflammation is associated with lymphocyte and macrophage infiltration, blood vessel proliferation, and fibrosis, often leading to destruction of the tissue [4,5]. During the course of inflammatory diseases, the immune system over-regulates the cytokine production in a feedback loop leading to increased levels that result in inflammation. Cytokines, referred to as interleukins (IL), chemokines, and growth factors, are small proteins that are secreted by a variety of cell populations such as resident innate or adaptive immune cells, infiltrating inflammatory cells, or epithelial cells [6]. Thereby, epithelial cells in inflamed regions express pro-inflammatory cytokines such as IL-1ß, IL-6, IL-8, and tumor necrosis factor alpha (TNF-α). This release leads to the activation of immune cells that accumulate at inflamed sites and to the production and release of further cytokines [7].

The pro-inflammatory reactions after tissue injury are mainly triggered by IL-1, which is associated with the innate immune system. Additionally, IL-1 is involved in the expression of genes encoding IL-8 [8,9,10]. Both IL-1α and IL-1ß together with IL-8 belong to the primary and secondary inflammatory cytokines. IL-8 induces the directional migration of immunocompetent and primary effector cells (i.e., polymorphonuclear neutrophils, monocytes and T cells) leading to accumulation of leukocytes at the site of inflammation [11,12,13]. Another pro-inflammatory cytokine that triggers the release of IL-8 is TNF-α. It is significantly involved in all inflammatory processes and its release leads to vasodilation, accompanied by redness, swelling, and increased vascular permeability [4,8]. In addition to systemic immune reactions, an imbalance of the bacterial environment in the mouth can also lead to inflammatory processes. Thereby, bacteria, and certain surface molecules of bacteria such as lipoproteins or lipopolysaccharides (LPS) interact with pathogen recognition receptors also referred to as toll like receptors (TRL). This binding initiates a series of events, which also lead to the production of cytokines such as IL-8 [3,14,15]. Besides IL-8, other biomarkers for the assessment of inflammation in the oral cavity are reactive oxygen species (ROS), IL-6, and prostaglandin E_2_ (PGE_2_) [14,15].

In-vivo studies remain the gold standard to study processes in oral pathogenesis. However, such studies are expensive, ethically questionable, and vary between individuals. Therefore, in-vitro cell systems that mimic in-vivo conditions are becoming increasingly important. Cell lines used to mimic the epithelial lining mucosa include primary cells, immortalized cell lines, and human tumor-derived cell lines such as HO-1-u-1 cells and TR146 cells [16,17,18]. TR146 cells are derived form a neck node metastasis of a buccal cell carcinoma [19]. Nielson et al. and Jacobsen et al. carefully characterized the cell line and showed that they form a confluent stratified non-keratinized squamous cell layer [20,21]. Thereby, TR146 cells are connected mainly via desmosomes; values for the transepithelial electric resistance (TEER) found in the literature vary but are rather low compared to the intestinal epithelial [22,23]. Further work by Lin at al. showed that when culture conditions of TR146 cells were optimized by e.g., adjusting the cell culture medium, barrier properties could be improved, making the model suitable for (drug) transport and permeability studies [24]. Apart from drug permeability, TR146 cells are also used to study nanoparticle interactions and cytotoxic effects. Teubl et al. showed that nanoparticle uptake is size and charge dependent [25]. Moreover, TR146 cells are used to monitor the inflammatory potential of a drug or device. For example, Schmalz et al. showed that TR146 cells release IL-8 upon stimulation with e.g., Ni dental casting alloy [11]. Soomro et al. treated TR146 cells with neutrophil elastase, which is high in patients suffering from Behcet’s disease. They found that IL-8 and TNF-α levels were not affected and IL-1ß production was slightly increased [26]. Further studies showed that TNF-α induced IL-8 production already at concentrations starting at 10 ng/mL as well as CCL20, a Th17 chemokine in periodontal lesions [27]. A commercially available 3D model based on TR146 cells is the reconstituted human oral epithelial model (ROHE). Recently, it has been used to study cellular response to *C. albicans* infections. In the studies, a fivefold increased expression of TNF-α was detected after *C. albicans* infection [17,18].

Although a large number of studies have been performed examining TR146 cells for drug transport, interaction studies, and monitoring the inflammatory potential of drug candidates, there is little information whether and how they change during inflammatory processes [28,29]. However, these changes could affect the function of topically applied drug delivery systems such as nanocarriers, and therefore must be considered when developing a formulation.

The aim of this study was to investigate morphological cell alterations induced by pro-inflammatory cytokines and LPS and examine their effects on nanoparticle interactions. The concentrations of cytokines and LPS used were selected based on physiological inflammation data from the literature [30,31,32]. Since the concentrations did not induce an inflammatory event in-vitro, they were carefully increased until IL-8 release was induced without causing cell death. The endpoints used were cell viability, cell membrane integrity, the production of ROS, and IL-8 release. Based on the results, morphological cell changes, i.e., impact on cell-cell connections, lipid distribution in the membrane, membrane charge, and re-organization of the cytoskeleton were evaluated. Finally, uptake mechanisms of pharmaceutically relevant nanoparticles, so-called nanostructured lipid carriers (NLC), were examined in treated and untreated cells to determine the consequences of barrier alterations on lipid drug delivery systems.

## 2. Materials and Methods

### 2.1. Materials

Human buccal TR146 cells were obtained from Imperial Cancer Research Technology (London, UK). IL-1α human, LPS from Escherichia coli O55:B5, MEM Non-essential Amino Acid Solution (100×; NEAA), Tween 80, oil red o, hydrogen peroxide (H_2_O_2_), ethylenediaminetetraacetic acid (EDTA), Bovine Serum Albumin (BSA), Dynasore hydrate, Chlorpromazine hydrochloride, Genistein, and 5-(N-Ethyl-N-isopropyl)amiloride (EIPA) were purchased from Sigma-Aldrich (Munich, Germany). Dihydroethidium (DHE), Alexa Fluor 488 Phalloidin, Hoechst 33342, IL-1ß, human protein, recombinant, Gibco™ and TNF-α Recombinant Human Protein were obtained from Thermo Fisher Scientific (Vienna, Austria). Palmitic acid (Merck KgaA, Darmstadt, Germany) and oleic acid (Croda GmbH, Nettetal, Germany) were used for the preparation of NLC. Dulbecco’s Modified Eagle’s medium (DMEM), fetal bovine serum (FBS), penicillin streptomycin (Penstrep), Hank’s Balanced Salt Solution (HBSS), phosphate buffered saline (PBS; pH 7.4), and 0.25% trypsin-ethylenediaminetetraacetic acid (trypsin-EDTA) from Gibco, Life Technologies Corporation (Painsley, UK) were used for all cell culture experiments. HyClone (DMEM without phenol red) was obtained from GE Healthcare Life Sciences (Logan, UT, USA). Ultra purified water (i.e., Milli-Q^®^-water (MQ-water), Millipore S.A.S., Molsheim, France) was used for all experiments.

### 2.2. Methods

#### 2.2.1. Cell Culture

TR146 cells were cultured in DMEM supplemented with 10% FBS, 1% Penstrep, and 1% NEAA at 37 °C in a humidified atmosphere with 5% CO_2_. Sub-cultivation of confluent cells was performed on a weekly basis using trypsin-EDTA. The medium of the cells was changed every second to third day. Supplemented serum-free DMEM without phenol red was used for all experiments.

#### 2.2.2. Toxicity Studies

TR146 cells were seeded (2 × 10^4^ cells/100 µL) in 96-well plates (Greiner Bio-One GmbH, Frickenhausen, Germany) and cultured for 24 h. Subsequently, the medium was replaced by IL-1α, IL-1β, LPS, and TNF-α diluted with serum-free DMEM and incubated for 24 h (n = 6). For each stimulant, different concentrations were used: (i) IL-1α (100–800 ng/mL), (ii) IL-1β (100–800 ng/mL), (iii) LPS (300–70,000 ng/mL), and (iv) TNF-α (100–800 ng/mL). To determine the cell viability, a CellTiter 96 Aqueous Non-Radioactive Cell Proliferation Assay (Promega Corporation, Madison, WI, USA) was used according to the manufacturer’s instructions. After an incubation time of 4 h, the absorbance was measured at 490 nm using a UV-/VIS-plate reader (Fluostar Galaxy, BMG Labtech GmbH, Ortenberg, Germany). Untreated, blank-corrected wells represented 100% viability. The membrane integrity of TR146 cells was investigated by determining the lactate dehydrogenase (LDH) release using a CytoTox-ONE Homogeneous Membrane Integrity Assay (Promega) according to the manufacturer’s instructions. The fluorescence signal was measured at an excitation wavelength of 560 nm and an emission wavelength of 590 nm with a UV-VIS-plate reader (Fluostar Galaxy, BMG Labtech). Control wells representing 100% LDH release were treated with 2 µL of a lysis solution and all data were blank corrected.

#### 2.2.3. Generation of Reactive Oxygen Species

To determine the generation of ROS, TR146 cells were seeded in 96-well plates (Nunc™ MicroWell™ 96-Well Optical-Bottom Plates with Polymer Base; Thermo Fisher Scientific) with a seeding density of 2 × 10^4^ cells/100 µL and incubated for 24 h. After 24 h the medium was replaced by (i) IL-1α (i.e., 100, 200, 300 and 400 ng/mL), (ii) IL-1β (i.e., 100, 200, 300, and 400 ng/mL), (iii) TNF-α (i.e., 100, 200, 300 and 400 ng/mL), and (iv) LPS (i.e., 300, 1000, 2000, and 10,000 ng/mL) diluted in serum-free DMEM. H_2_O_2_ diluted with serum-free DMEM to reach a final concentration of 200, 400, and 800 µM was used as reference for the maximum ROS production. Untreated wells served as control for viable cells. After an incubation time of 24 h, 100 µL of DHE diluted in serum-free DMEM at a concentration of 10 µM was added to each well and incubated for 4 h. Subsequently, the fluorescence was measured at an excitation wavelength of 544 nm and an emission wavelength of 612 nm using a Fluostar OPTIMA (BMG Labtech).

#### 2.2.4. Interleukin-8 Release

For the determination of the IL-8 release, TR146 cells were seeded in 24-well plates (Greiner Bio-One GmbH, Frickenhausen, Germany) with a seeding density of 4 × 10^4^ cells/well and cultured for 1 week. Five-hundred microliters of IL-1α (i.e., 100, 200, 300 and 400 ng/mL), IL-1β (i.e., 100, 200, 300, and 400 ng/mL), LPS (i.e., 300, 1000, 2000, and 10,000 ng/mL), and TNF-α (i.e., 100, 200, 300, and 400 ng/mL) diluted in serum-free DMEM were applied and incubated for 24 h. Untreated wells served as control. After 24 h, the supernatants of the cells were collected and stored at −80 °C (ULUF 65, Arctiko, Esbjerg N, Denmark) until further use. The IL-8 release of the samples was measured using enzyme-linked immune assays (ELISA) (n = 4). Human IL-8 ELISA sets (BD OptEIA™) from BD Biosciences (Vienna, Austria) were used according to the manufacturer’s instruction at different concentrations. The absorbance of the samples was measured at 450 nm using a SPECTRAmax (Molecular Devices, San Jose, CA, USA). The IL-8 release was calculated using the standard curve prepared for each ELISA.

#### 2.2.5. Determination of Cell Proliferation under Inflamed Conditions

For cell proliferation studies, TR146 cells were seeded in 96-well plates with a seeding density of 5 × 10^3^ cells/well and cultured for 24 h. Subsequently, the medium was replaced by IL-1ß (i.e., 100, 200, 300, and 400 ng/mL) and TNF-α (i.e., 100, 200, 300, and 400 ng/mL) diluted with DMEM. After an incubation time of 1, 3, and 7 days, the cell proliferation was investigated using a CellTiter 96 Aqueous Non-Radioactive Cell Proliferation Assay (Promega) according to the manufacturer’s instructions. After an incubation time of 4 h, the absorbance was measured at 490 m using a UV-/Vis plate reader (Fluostar Galaxy, BMG Labtech). Untreated wells were used as control and all wells were blank corrected.

#### 2.2.6. Visualization of Phosphatidylserine Translocation

TR146 cells were seeded in 8-well chamber slides (ibidi GmbH, Martinsried, Germany) with a seeding density of 2 × 10^4^ cells/300 µL and incubated for 2–3 days. Subsequently, medium was removed, (i) IL-1ß (i.e., 300, 400 and 800 ng/mL) and (ii) TNF-α (i.e., 200, 300 and 400 ng/mL) diluted in serum-free DMEM were added and incubated for 24 h. After 24 h, cells were washed twice with 1x Annexin binding buffer (ABB) of Vybrant Apoptosis Assay Kit #2 (Molecular Probes™, Thermo Fisher Scientific). Five microliters of Annexin V-Alexa Fluor 488 (staining phosphatidylserine (PS) exposing cells) and 2 µL propidium iodide (PI, staining DNA of necrotic cells) in 300 µL ABB were added and incubated at room temperature in the dark for 15 min. To remove unbound Annexin V-Alexa Fluor 488, cells were washed twice with ABB and covered with ABB. For imaging, a Leica DMI6000 B with IMC in connection with a Leica DFC360 FX camera and AF 6000 software was used. In addition to the brightfield transmission, the green fluorescence of Annexin V-Alexa Fluor 488 was measured with an excitation wavelength of 488 nm and an emission wavelength of 530 nm. For the red spectral region (PI), an excitation wavelength of 536 nm and an emission wavelength of 617 nm were used. Exposure time, intensity, and gain were fixed for all measurements.

#### 2.2.7. Visualization of Cell Morphological Changes

For the visualization of morphological changes, TR146 cells were seeded in 24-well plates (glass bottom, Porvair Sciences, Wrexham, UK) with a seeding density of 4 × 10^4^ cells/well and incubated for 1 week. Subsequently, the medium was replaced by IL-1β (i.e., 300 and 400 ng/mL) and TNF-α (i.e., 200 and 300 ng/mL) diluted in serum-free DMEM and incubated for 24 h. Untreated cells served as control and were incubated with serum-free DMEM. After incubation, the dilutions were removed, and cells were washed twice with PBS. The cytoskeleton of the cells was stained with Alexa Fluor 488 Phalloidin and the nuclei were counterstained with Hoechst 33342. Images were acquired with a LSM 510 Meta confocal laser scanning microscope (cLSM, Carl Zeiss GmbH, Vienna, Austria) equipped with ZEN2008 software package. Alexa Fluor 488 Phalloidin dyed cytoskeleton was detected at an excitation wavelength of 488 nm using a BP 505-550 nm bandpass detection for the green channel. Hoechst stained nuclei were visualized at 405 nm excitation wavelength with a BP 420–480 nm bandpass detection for the blue channel. Images of randomly chosen areas of the cell monolayers were captured via cLSM. Z-stacks were acquired, and virtual radial sections were documented.

#### 2.2.8. Visualization of Cell–Cell Contacts

Immunofluorescence analysis of goat anti-mouse immunoglobulin 1 (IgG1) cross-adsorbed secondary antibody, Alexa Fluor 568 conjugate (secondary antibody, Thermo Fisher Scientific, Waltham, MA, USA) was performed using TR146 cells, which were seeded in 24-well plates (4 × 10^4^ cells/well) and incubated for 1 week. The medium was replaced by (i) IL-1ß (i.e., 300 and 400 ng/mL) and (ii) TNF-α (i.e., 200 and 300 ng/mL) diluted in serum-free DMEM and incubated for 24 h. Untreated cells served as control and were incubated with serum-free DMEM. After an incubation time of 23 h, EDTA diluted with serum-free medium to reach a final concentration of 0.25 mM was added to the control wells and incubated for 1 h. EDTA was used as control for cell destabilization. After a washing, fixation and blocking procedure according to the manufacturer’s instructions, cells were labelled with the primary antibody (i.e., mouse anti-Desmoglein 3 Monoclonal Antibody, Thermo Fisher Scientific, Waltham, MA, USA) diluted with PBS to a final concentration of 4 µg/mL for 3 h at room temperature. Mouse IgG1 kappa isotype control (Thermo Fisher Scientific, Waltham, MA, USA) was used as negative control and was treated the same as the primary antibody. Moreover, PBS was added to control wells, which were not treated with the primary antibody. After 3 h, cells were washed and incubated with the secondary antibody (i.e., 4 µg/mL diluted with PBS containing 0.2% BSA) for 45 min. Images were acquired with cLSM. Goat anti-mouse IgG1 cross-adsorbed secondary antibody, Alexa Fluor 568 conjugate was detected at 543 nm excitation wavelength using a LP 560 nm long pass detection for the red spectral region.

#### 2.2.9. Preparation of Nanostructured Lipid Carriers via High Pressure Homogenization

Oil red o labeled NLC were prepared according to Tetyczka et al. [33,34]. Briefly, oil red o (2% *w*/*w*; % with regard to the lipid phase) was mixed with palmitic acid and oleic acid (10% *w*/*w*; ratio 9:1 palmitic acid and oleic acid) and heated to 70 °C. Subsequently, an aqueous stabilizer solution (i.e., 2% *w*/*w* Tween 80) that was heated to the same temperature was added. The mixture was stirred at 8000 rpm for 1 min with an Ultra Turrax (Ultra Turrax T25, Janke & Kunkel, IKA^®^-Labortechnik, IKA^®^-Werke GmbH & Co. KG, Staufen, Germany). The obtained pre-suspension was transferred into a high-pressure homogenizer (Panda 2K, NS1001L Spezial, GEA Niro Soavi, Lübeck, Germany), which is equipped with a water jacket to control the temperature. The system temperature was kept constant at 70 °C. The suspensions were homogenized running five cycles at 500 bars. Finally, the suspensions were transferred into glass vials and cooled in an ice-bath for 30 min. Blank NLC were produced without the addition of oil red o. If not otherwise stated, all concentrations were given with regard to the total formulation amount.

#### 2.2.10. Particle Size and Zeta Potential

Size analysis was conducted by Photon Correlation Spectroscopy (PCS) using a Zetasizer Nano ZS (Malvern Instruments, Malvern, UK), which is equipped with a 532 nm laser. The measurements were performed at a measurement angle of 173° (backscatter) at 25 °C with an equilibration time of 30 s. Prior to each measurement, samples were diluted with MQ-water. The refractive indices (RI) of the particles and MQ-water were set to 1.4381 (i.e., palmitic acid) and 1.3325, respectively. PCS yields the polydispersity index (PdI) as a measure of the width of the particle size distribution and the hydrodynamic diameter (z-average).

The zeta potential was investigated via Laser-Doppler-Micro-Electrophoresis coupled with PCS (Zetasizer Nano ZS, Malvern Instruments) using a scattering angle of 173° at 25 °C. The calculation was performed according to the Helmholtz-Smoluchovski equation [35]. Prior to each measurement, samples were diluted with zeta-water (i.e., MQ-water adjusted with 0.9% (*v*/*v*) sodium chloride solution to a pH of 5.5–6 and a conductivity of 50 µS/cm).

#### 2.2.11. Interaction Studies of TR146 Cells with Nanostructured Lipid Carriers

To determine potential effects of blank NLC on the generation of ROS, experiments were conducted. Briefly, blank NLC in concentrations of 500 and 750 µg/mL were applied on TR146 cells, incubated with 10 µM DHE for 4 h and the fluorescence was measured at an excitation wavelength of 544 nm and an emission wavelength of 612 nm using a Fluostar OPTIMA (BMG Labtech). Subsequently, IL-8 release of TR146 cells treated with blank NLC (i.e., 500 and 750 µg/mL) for 4 h was investigated using ELISA sets from BD Biosciences (Vienna, Austria). After measuring the absorbance of the samples at 450 nm (SPECTRAmax, Molecular Devices, San Jose, CA, USA), the IL-8 release was calculated using the standard curve prepared for each ELISA. Moreover, the impact of blank NLC (i.e., 500 and 750 µg/mL incubated for 4 h) on PS translocation, morphological changes, and cell–cell contacts was determined. For PS translocation studies, a Vybrant Apoptosis Assay Kit #2 from Molecular Probes™ was used according to the manufacturer’s instructions. Visualization of the samples was performed as described above (Section 2.2.6). Morphological changes were detected via cLSM. For this, the cytoskeleton of the cells was stained with Alexa Fluor 488 Phalloidin and the cell nuclei were counterstained with Hoechst. Immunofluorescence analysis was used to visualize cell–cell contacts. The experiments were conducted as described in Section 2.2.8 and images were acquired using cLSM.

#### 2.2.12. Qualitative and Semi-Quantitative Nanostructured Lipid Carriers Uptake Studies

TR146 cells were seeded in 24-well plates with a seeding density of 4 × 10^4^ cells/well and incubated for 1 week. Next, cells were incubated with IL-1ß (i.e., 100, 200, 300 and 400 ng/mL) and TNF-α (i.e., 100, 200, 300 and 400 ng/mL) diluted with serum-free DMEM for 24 h. After incubation, medium was removed and replaced by oil red o labeled NLC dispersions diluted with serum-free DMEM to reach final concentrations of 500 and 750 µg/mL and incubated for 4 h. Untreated cells were used as control and were incubated with serum-free DMEM or with particle dispersions (i.e., 500 and 750 µg/mL). After 4 h, the dispersions were removed and the cells were washed with PBS. For qualitative and semi-quantitative uptake studies, the cytoskeleton of TR146 cells was stained with Alexa Fluor 488 Phalloidin and the nuclei were counterstained with Hoechst 33342. Images were acquired with a LSM 510 Meta cLSM equipped with ZEN2008 software package. Particles were detected at 633 nm excitation wavelength using a LP 650 nm long pass detection for the red channel. Images of randomly chosen areas of the cell monolayers were captured via cLSM. Z-stacks were acquired, and virtual radial sections were documented. The semi-quantitative particle uptake was further evaluated using the Nikon Software NIS-Elements (Nikon, Germany). For this, a defined number of cells and their respective surface area was calculated. Further, the area of the particles (µm^2^) was assessed. Based on this, the percentage of particles taken up by the cells was calculated.

#### 2.2.13. Determination of Active and Passive Transport Mechanisms

TR146 cells were seeded in 24-well plates with a seeding density of 4 × 10^4^ cells/well and incubated for 1 week. Next, cells were incubated with IL-1ß (i.e., 100, 200, 300, and 400 ng/mL) and TNF-α (i.e., 100, 200, 300, and 400 ng/mL) diluted with serum-free DMEM for 24 h. After incubation, medium was removed and exchanged with Dynasore (i.e., 200 µM), Chlorpromazine (i.e., 20 µM), Genistein (i.e., 300 µM), and EIPA (i.e., 0.5 µM) with the respective inflammatory cytokines diluted in serum-free DMEM and incubated for 40 min at 37 °C. Subsequently, oil red o labeled NLC dispersions at concentrations of 500 and 750 µg/mL were added and incubated for 4 h. Untreated cells were used as control and were incubated with serum-free DMEM or with inhibitors and particle dispersions (i.e., 500 and 750 µg/mL) for 4 h at 37 °C. Moreover, cells were incubated with particle dispersions (i.e., 500 and 750 µg/mL) at 4 °C for 4 h. After 4 h, the dispersions were removed, and the cells were washed with PBS. The cytoskeleton of TR146 cells was stained as described in Section 2.2.7 and Section 2.2.12.

#### 2.2.14. Statistical Analysis

The results were presented as mean values ± standard deviation (SD). Statistical analysis (student’s t-test) was performed and data were compared with the respective control. Differences were considered to be significant at a level of *p* ≤ 0.05 (*), *p* ≤ 0.01 (**), and *p* ≤ 0.001 (***).

## 3. Results

### 3.1. Toxicity Studies

**Figure 1:**
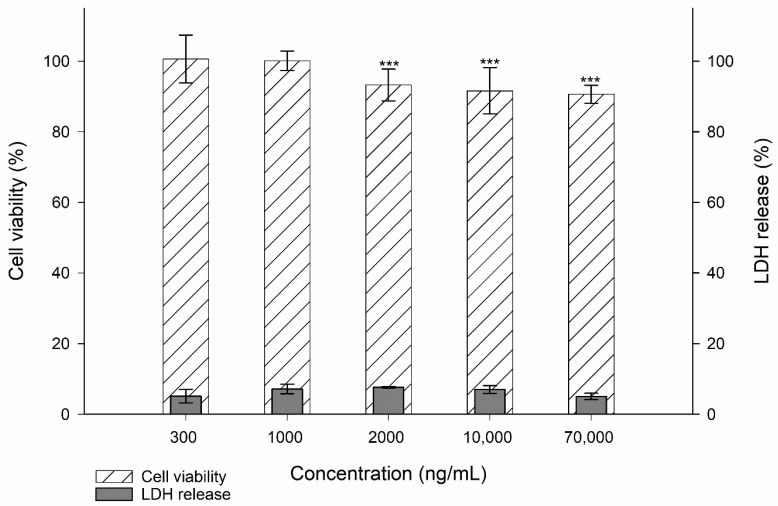
Cell viability and membrane integrity (i.e., LDH release) of TR146 cells treated with 300–70,000 ng/mL LPS. The percentages given (% ± SD) refer to the corresponding controls. Student’s *t*-test was used for statistical analysis.Significant differences compared to the control are marked as ***, which corresponds to a *p* value ≤ 0.001.

**Figure 2:**
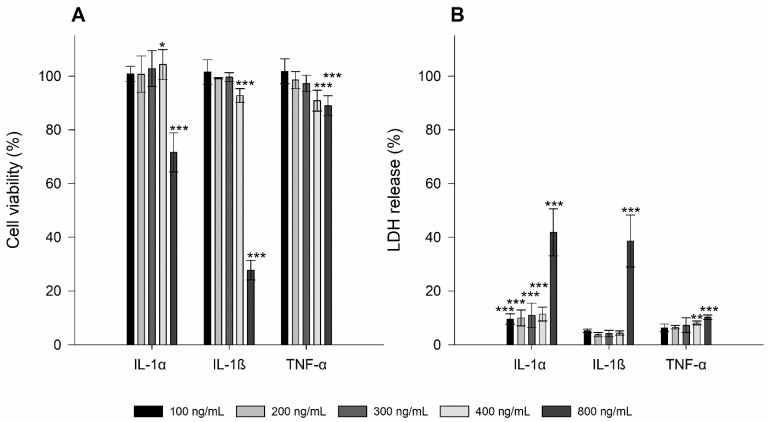
Cell viability (**A**) and membrane integrity (i.e., LDH release; (**B**)) of TR146 cells treated with 100–800 ng/mL IL-1α, IL-1ß, and TNF-α. The percentages given (% ± SD) refer to the corresponding controls. Student’s *t*-test was used for statistical analysis. Significant differences compared to the control are marked as *, which correspond to a *p* value ≤ 0.05, **, which corresponds to a *p* value ≤ 0.01 and ***, which corresponds to a *p* value ≤ 0.001.

To determine if stimulants (i.e., LPS, IL-1α, IL-1ß, and TNF-α) alter TR146 cells in a concentration-dependent manner, the cell viability and the membrane integrity were investigated and compared to a cell control. All results are presented in Figure 1 and Figure 2.

For LPS, which was tested at concentrations of 300–70,000 ng/mL, a significant decrease in cell viability was observed starting at a concentration of 2000–70,000 ng/mL (i.e., *p* ≤ 0.001 ***). However, the measured viability was still above 90%. The membrane integrity was comparable to the cell control with no significant alterations in the LDH release. IL-1α at concentrations from 100 to 400 ng/mL showed good comparability to the cell control. The highest concentration of 800 ng/mL significantly decreased the cell viability to 71.61 ± 7.25% (i.e., *p* ≤ 0.001 ***). The LDH release at the lowest concentration was 9.56 ± 1.95% and increased with higher concentration to 41.81 ± 8.75% (i.e., *p* ≤ 0.001 ***).

Similar to IL-1α, no cytotoxic effects were found at IL-1ß concentrations between 100 and 300 ng/mL. Starting from a concentration of 400 to 800 ng/mL, a significant decrease in cell viability to 92.80 ± 2.57% and 27.74 ± 3.64%, respectively, was obtained (i.e., *p* ≤ 0.001 ***). At a concentration of 800 ng/mL, the LDH release increased to 38.64 ± 9.66% (*p* ≤ 0.001 ***), which correlates with a decreased cell viability.

The lowest concentration of TNF-α (i.e., 100 mg/mL) neithered affect cell viability nor membrane integrity compared to the cell control. Similar to IL-1ß, a concentration of 400 ng/mL already triggered a significant loss in cell viability to 90.88 *** ± 3.91% followed only by a marginal decrease at a concentration of 800 ng/mL (i.e., 88.99 ± 3.73%, *p* ≤ 0.001 ***). These concentrations also affected the LDH release (i.e., 8.12 ± 0.69% and 10.25 ± 0.82% with *p* values of ≤0.01 ** and 0.001 ***, respectively).

### 3.2. Generation of Reactive Oxygen Species

The potential generation of ROS was assessed by the ROS-driven conversion of the non-fluorescent DHE to the fluorescent ethidium. The production of these oxygen species occurs due to an incomplete reduction of molecular oxygen leading to the formation of superoxide anions ($O_{2}^{.-}$), hydrogen peroxide (H_2_O_2_), and highly reactive hydroxyl radicals (HO·) [36]. The cell control was used as standard to calculate the x-fold increase of ROS and thus, increase of DHE. Four concentrations were tested on the basis of their safety according to the results of the previous cell viability and membrane integrity tests. For LPS, the highest concentration, i.e., 70,000 ng/mL, and for IL-1α, IL-1ß, and TNF-α the concentration of 800 ng/mL each was excluded.

**Figure 3:**
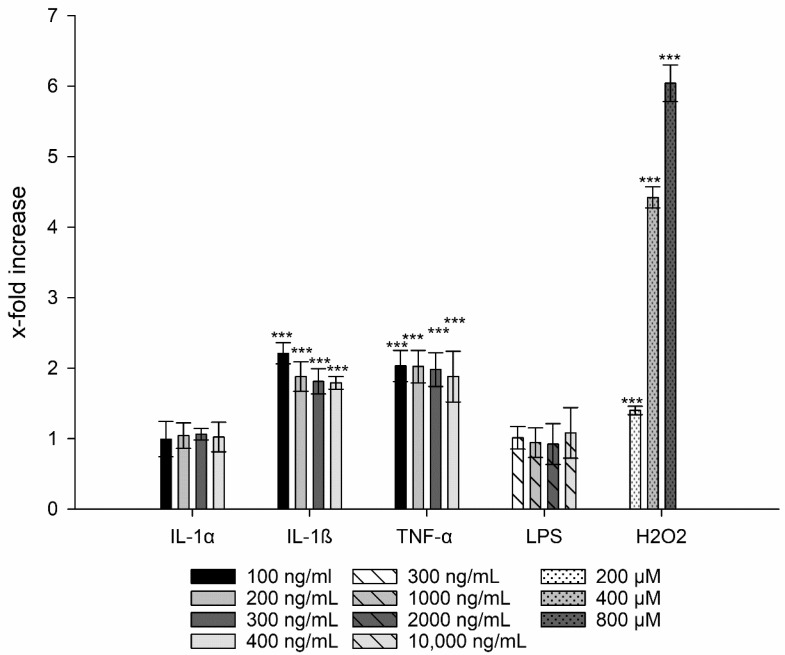
Generation of ROS after treatment with IL-1α, IL-1ß, LPS, and TNF-α compared to untreated cells. H_2_O_2_ was used as positive control. Student´s *t*-test was used for statistical analysis. Significant differences compared to the control are marked as ***, which corresponds to a *p* value ≤ 0.001.

After treatment of TR146 cells with LPS and IL-1α, the production of ROS was comparable with the untreated control (Figure 3). By contrast, the addition of H_2_O_2_ (200 to 800 µM) showed a significant increase in ROS from 1.40 ± 0.06 to 6.04 ± 0.26 (*p* ≤ 0.001 ***) in a concentration-dependent manner. Similar to the positive control, treatment with IL-1ß at concentrations of 100–400 ng/mL showed a significant increase in ROS generation; the same results were found for TNF-α (approx. 2-fold, *p* ≤ 0.001 ***). Taking into account the standard deviations, no differences were found in ROS production within the tested concentration range.

### 3.3. Interleukin-8 Release

IL-8 is one of the most important chemokines in inflammatory processes and is released by different cell types. This release occurs after stimulation of epithelial cells with cell components of microorganisms, such as LPS and endogenous mediators including all cytokines or cellular stress [12,13]. A 3-fold (or higher) production of IL-8 can be considered a significant increase, and thus a trigger for inflammation [11,37].

**Figure 4:**
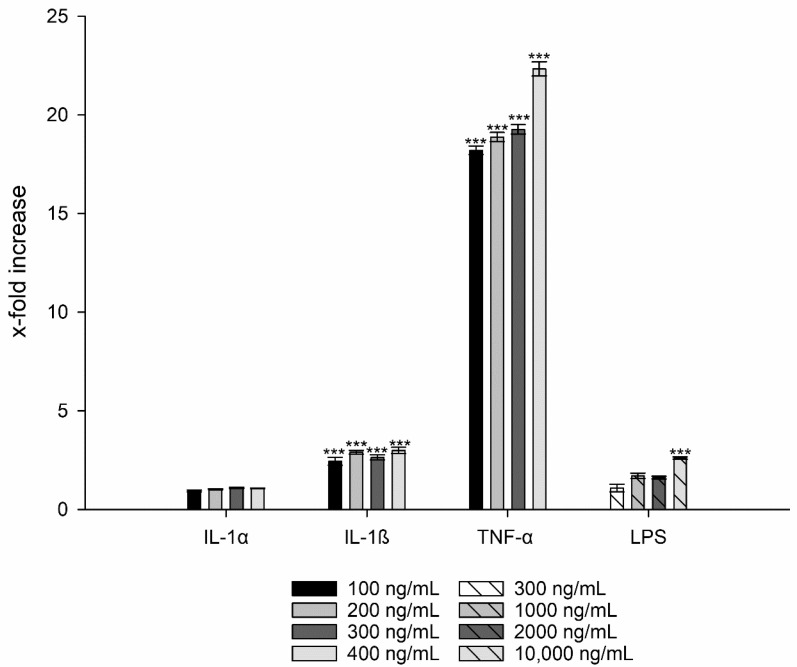
IL-8 release of TR146 cells after treatment with LPS, IL-1α, IL-1ß, and TNF-α compared to untreated cells. Student´s *t*-test was used for statistical analysis. Significant differences compared to the control are marked as ***, which corresponds to a *p* value ≤ 0.001.

For LPS a 2.6-fold and for IL-1α a 1.1-fold increase in IL-8 production could be observed for TR146 cells (i.e., 2.61 ± 0.06, *p* ≤ 0.01 **) regardless of the concentration (Figure 4). Hence, both LPS and IL-1α were excluded as potential candidates to induce inflammation.

In contrast, IL-1ß showed a 3-fold increased IL-8 release at a concentration of 400 ng/mL (i.e., 2.99 ± 0.16, *p* ≤ 0.001 ***) (Figure 4). An even stronger effect was observed with TNF-α, which showed an 18-fold increase in IL-8 production at the lowest concentration (100 ng/mL) compared to the untreated cell control. The highest values were measured at a concentration of 400 ng/mL with a 22-fold increase in IL-8 (i.e., 22.34 ± 2.09, *p* ≤ 0.001 ***).

### 3.4. Cell Proliferation under Inflamed Conditions

**Figure 5:**
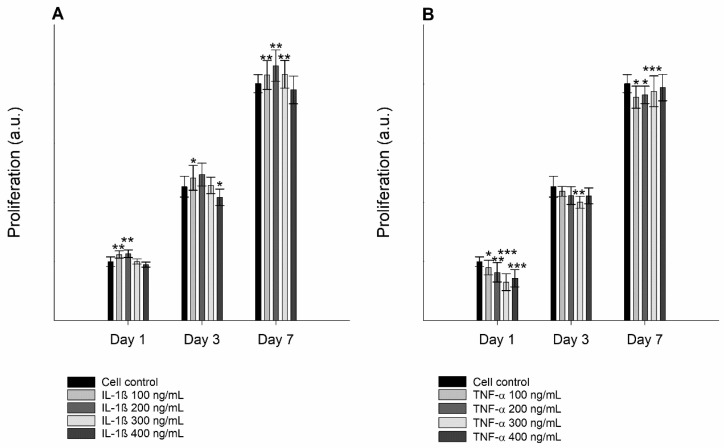
Proliferation of untreated TR146 cells and cells treated with 100–400 ng/mL IL-1ß (**A**) and TNF-α (**B**) over a period of 1, 3, and 7 days using a CellTiter 96 Aqueous Non-Radioactive Cell Proliferation Assay. Student´s *t*-test was used for statistical analysis. Significant differences compared to the control are marked as *, which corresponds to a *p* value ≤ 0.05, **, which corresponds to a *p* value ≤ 0.01 and ***, which corresponds to a *p* value ≤ 0.001.

Due to the morphological alterations, a change in the proliferation rate of cells treated with inflammatory mediators was expected. The cell proliferation studies showed that TR146 cells treated with both IL-1ß and TNF-α revealed a moderate to significantly reduced growth rate (Figure 5).

### 3.5. Visualization of Phosphatidylserine Translocation

**Figure 6:**
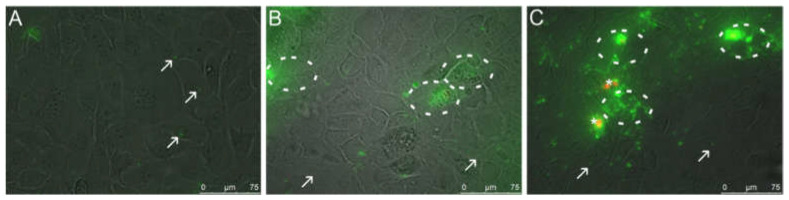
Fluorescence microscopic images (overlay of bright field and fluorescence channels) of untreated TR146 cells (**A**) and cells treated with 400 ng/mL IL-1ß (**B**) and 300 ng/mL TNF-α (**C**). Circles indicate specific binding of Annexin V-Alexa Fluor 488 (green) to PS on the outside of the cells. Arrows show cell blebbing (green spots) and stars indicate PI stained necrotic cells (red).

Inflammatory processes lead to changes in the structure of the cell membrane, more specifically to PS translocation from the inner to the outer leaflet. The results of the negative (untreated) control (Figure 6A) showed no PI positive necrotic cells, assuring the integrity of the cell layer. Only small PS exposing blebs but no PS exposing clusters were found, indicating that only a small amount of PS was present in the outer leaflet of the cell membrane. For both IL-1ß and TNF-α treated cells, increased PS exposure was detected in the outer leaflet of the cell membrane after 24 h of incubation, indicating the induction of apoptosis. After incubation with IL-1ß, no PI positive necrotic cells were seen at concentrations of 400 ng/mL (Figure 6B). Annexin V-Alexa Fluor 488 selectively bound to PS on the surface of IL-1ß treated cells and was localized in clusters on the cell membrane, visualized by a strong increase in fluorescence intensity. Moreover, small PS exposing blebs were visible. At higher IL-1ß concentrations of 800 ng/mL, cell damage and further cell loss upon washing steps was clearly visible by microscopic inspection accompanied with a decrease in PS signaling (Supplementary Figure S1A). Therefore, the concentration of 800 ng/mL was re-evaluated to visualize the apoptotic and necrotic state of TR146 cells, which was determined by MTS and LDH assay. Results showed that the cell viability decreased to 27.74 ± 3.64% and the LDH increased to 38.64 ± 9.66%, respectively. The results showed that increasing the IL-1ß concentration to 800 ng/mL triggered cell death.

A concentration of 300 ng/mL of TNF-α also induced stress-related blebs (Figure 6C). Figure 6C showed a “granular” green fluorescence, which indicates that at this concentration, TNF-α mainly induces apoptosis indicated by translocation of PS to the outer leaflet of the membrane. Only a minor population of PI positive necrotic cells were found, suggesting that TNF-α treated cells did not mainly undergo necrosis. Increasing the TNF-α concentration to 400 ng/mL significantly increased the uptake of PI and exposure of PS (Supplementary Figure S1B). Thus, this concentration was excluded from further experiments.

### 3.6. Visualization of Cell Morphological Changes

**Figure 7:**
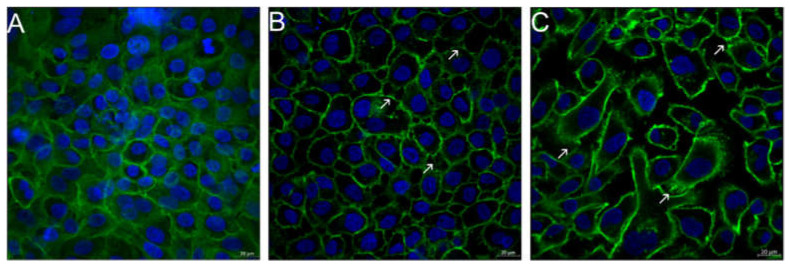
Fluorescence microscopic images of untreated TR146 cells (**A**) and TR146 cells treated with 400 ng/mL IL-1ß (**B**) and 300 ng/mL TNF-α (**C**). Cell nuclei were stained with Hoechst (blue) and the cytoskeleton was stained with Alexa Fluor 488 Phalloidin (green). Arrows show the formation of stress fibers.

Visualization of the cell structure of TR146 cells after incubation with the potential inflammatory triggers IL-1ß and TNF-α, revealed inflammation-induced re-organizations. In general, TR146 are epithelial cells with a characteristic squamous morphology, prominent cell nuclei and closed cell–cell contacts (i.e., desmosomes) at confluent conditions. After treating the cells with IL-1ß and TNF-α, a structural re-organization of the cytoskeleton was observed (Figure 7B,C). The cell nuclei were larger, and the cytoskeleton showed a disordered structure compared to the cell control (Figure 7A). Increased intercellular spaces and dense filamentous sections, so called stress fibers, were visible especially for TNF-α and to a lesser extent for IL-1ß. Stress fibers are composed of bundles of 10–30 actin filaments, which contain bipolar arrays of myosin II and consecutive α-actinin-foci. Depending on the cell type, cell region and cell development stage variations of the orientation of the filaments are possible [38,39].

### 3.7. Loss of Cell–Cell Contacts (Desmosomes)

The effects of inflammatory processes on cell–cell contacts (i.e., desmosomes) were visualized. The cell control (Supplementary Figure S2A) showed no red fluorescence, indicating intact intercellular junctions and a confluent cell layer. Moreover, no staining was observed in the negative control (data not shown), which ensures that no unspecific binding occurred. On the contrary, the positive control treated with EDTA, which led to complex formation with calcium ions and resulted in cell-junction opening, showed an increase in the red fluorescence (Supplementary Figure S2B). Incubating the cells with IL-1ß (i.e., 400 ng/mL; Supplementary Figure S2C) and TNF-α (i.e., 300 ng/mL; Supplementary Figure S2D) also showed a red fluorescence, implying a loss of cell–cell contacts. For both IL-1ß and TNF-α, the signal was higher compared to the positive control. In the case of TNF-α, it could also be shown that the cells were no longer present in a cluster but as isolated cells.

### 3.8. Nanostructured Lipid Carriers Interaction and Uptake Studies

**Figure 8:**
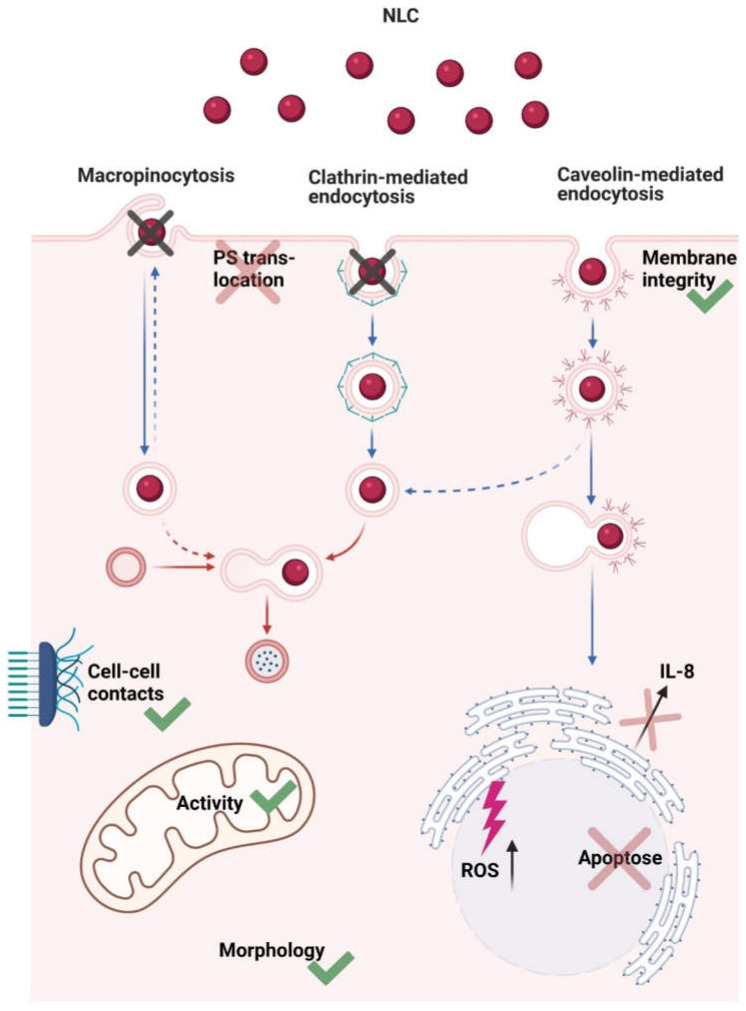
Schematic illustration describing NLC–cell interactions and uptake mechanism. Briefly, NLC were transported into the cell via caveolin-mediated endocytosis. Membrane integrity and mitochondrial activity were not affected; there was no translocation of PS, cell morphology was comparable to the control, and cell–cell-contacts remained intact. Apoptosis was not induced, there was no increase in IL-8 release, but ROS production was increased.

After particle preparation, blank NLC showed a z-average of 276.32 ± 2.49 nm (PdI values of 0.234 ± 0.023). Oil red o labeled NLC revealed a z-average of 293.44 ± 19.72 nm with a narrow particle size distribution (i.e., PdI values of 0.241 ± 0.042). The zeta potential of blank NLC and labeled NLC was −37.27 ± 0.82 mV and −39.16 ± 4.09 mV, respectively, indicating good physical stability [34]. Moreover, the particles remained stable after dispersion in DMEM (data not shown). They neither impacted mitochondrial activity nor membrane integrity of TR146 cells at concentrations of 500 and 750 µg/mL [33]. However, both NLC concentrations showed a significant increase in ROS production (i.e., 1.14 ± 0.09 (*p* ≤ 0.05 *) and 1.26 ± 0.12 (*p* ≤ 0.001 ***)). In contrast, no increase in IL-8 release was detected and levels were comparable to the control (i.e., 0.58 ± 0.19 (500 µg/mL) and 0.93 ± 0.53 (750 µg/mL)). This correlated with the PS translocation studies, as no differences from the untreated cells were evident. Similarly, no morphological changes were observed and cell–cell contacts remained intact (data not shown). A schematic overview of the results is shown in Figure 8.

**Figure 9:**
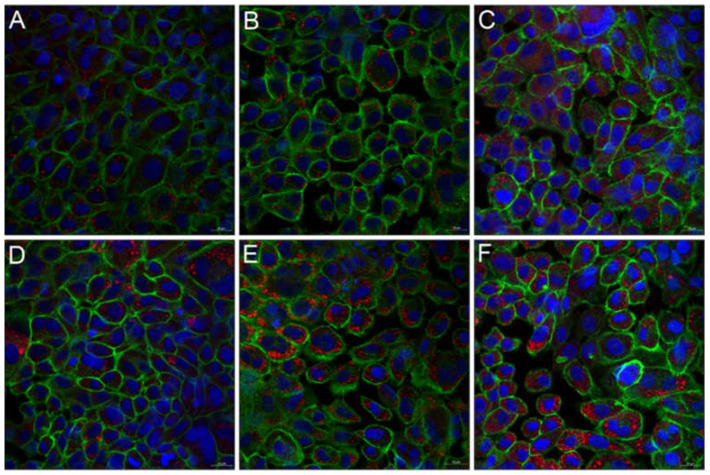
Fluorescence microscopic images of untreated TR146 cells (**A**,**D**) and TR146 cells treated with 400 ng/mL IL-1ß (**B**,**E**) and 300 ng/mL TNF-α (**C**,**F**). Particle uptake was performed with 500 µg/mL (**A**–**C**) and 750 µg/mL (**D**–**F**) NLC. Cell nuclei were stained with Hoechst (blue) and the cytoskeleton was stained with Alexa Fluor 488 Phalloidin (green). NLC were labeled with oil red o (red).

**Figure 10:**
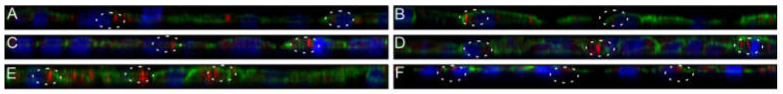
Fluorescence microscopic z-scans of untreated TR146 cells (**A**,**D**) and TR146 cells treated with 400 ng/mL IL-1ß (**B**,**E**) and 300 ng/mL TNF-α (**C**,**F**). Particle uptake was performed with 500 µg/mL (**A**–**C**) and 750 µg/mL (**D**–**F**) NLC. Cell nuclei were stained with Hoechst (blue) and the cytoskeleton was stained with Alexa Fluor 488 Phalloidin (green). The circles represent particle uptake/internalization into TR146 cells. NLC were labeled with oil red o (red).

After stimulating the cells with IL-1ß and TNF-α (i.e., 100–400 ng/mL, respectively), TR146 cells were incubated with 500 and 750 µg/mL particles. Thereby, the NLC uptake was dependent on the degree of inflammation, the cytokine used, and the particle concentration applied. IL-1ß and TNF-α concentrations of 400 and 300 ng/mL showed the highest IL-8 release and ROS production and induced morphological changes. Consequently, treatment with these cytokine concentrations resulted in the highest particle internalization. Figure 9 and Figure 10 show the concentration-dependent NLC uptake into untreated and cytokine-treated cells. Overall, it was found that particle uptake was higher in cytokine treated than in untreated cells. Depending on the applied NLC concentration, 2 or 11% of the particles were internalized by the untreated cells (Figure 9A,D and Figure 10A,D). Cell treatment with IL-1ß increased the uptake of NLC to a minor extent (i.e., 5 vs. 16%, Figure 9B,E and Figure 10B,E). By contrast, incubation with TNF-α led to a significant increase in NLC uptake to 17% and 39% (*p* ≤ 0.001 ***), respectively, which was confirmed by a strong increase in red fluorescence intensity (Figure 9C,F and Figure 10C,F).

**Figure 11:**
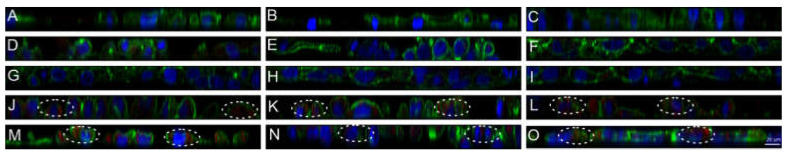
Fluorescence microscopic z-scans of untreated TR146 cells at 4 °C (**A**) and TR146 cells treated with 400 ng/mL IL-1ß and 300 ng/mL TNF-α at 4 °C (**B**,**C**). TR146 cells treated with Dynasore (**D**) and additionally with IL-1ß and TNF-α (**E**,**F**). TR146 cells treated with Genistein (**G**) and additionally with IL-1ß and TNF-α (**H**,**I**). TR146 cells treated with Chlorpromazine (**J**) and additionally with IL-1ß and TNF-α (**K**,**L**). TR146 cells treated with EIPA (**M**) and additionally with IL-1ß and TNF-α (**N**,**O**). Cell nuclei were stained with Hoechst (blue) and the cytoskeleton was stained with Alexa Fluor 488 Phalloidin (green). The circles represent particle uptake/internalization into TR146 cells. NLC were labelled with oil red o (red).

The uptake mechanisms involved, i.e., active and passive, were investigated using relevant conditions and inhibitors at concentrations that did not affect the cells viability. It was found that the uptake mechanism of NLC was independent of the conditions present. Cells incubated at 4 °C showed no NLC uptake regardless of whether inflammation was induced or not (Figure 8 and Figure 11A–C). After incubation with Dynasore, which inhibits caveolin and clathrin-mediated endocytosis, no particles were found in the cytoplasm of treated as well as untreated cells (Figure 11D–F). Similar results were obtained for Genistein, which exclusively inhibits caveolin-mediated endocytosis (Figure 11G–I). On the contrary, inhibition with Chlorpromazine, an inhibitor of the clathrin-mediated endocytosis, led to an increased internalization of NLC into both IL-1ß and TNF-α treated and untreated TR146 cells (Figure 11J–L). This suggests that caveolin-mediated endocytosis is the main route involved as the inhibition of macropinocytosis using EIPA also showed NLC uptake (Figure 8 and Figure 11M–O).

## 4. Discussion

Inflammation of the oral mucosa is dependent on the underlying disease and the respective inflammation cascade. The treatment is carried out either by local therapies such as antibacterial mouthwashes, or systemic therapies using highly potent pharmacological drugs [40]. Administration of these drugs may be orally, which can be challenging, especially for patients with swallowing difficulties due to xerostomia or hyposalivation [41]. In addition, fragile biologicals such as monoclonal antibodies, proteins and growth factors can be inactivated by the harsh conditions in the gastrointestinal tract. Parenteral administration is invasive and therefore associated with pain for the patients, unwanted side effects, and higher healthcare costs [42,43,44]. Therefore, topical administration of drug candidates directly to the oral mucosa is a promising strategy to achieve a fast therapeutic local effect and avoid additional side effects. In medication development, however, many studies do not take into account the changes in the barrier systems in the mouth caused, for example, by pro-inflammatory cytokines [45]. Accordingly, the potential of local drug delivery systems to transport the active ingredient to the site of action is often limited.

In this study, four inducers of inflammation, namely LPS, IL-1α, IL-1ß, and TNF-α were tested. To assess their potential in TR146 cells, parameters including ROS and IL-8 were used to classify whether or not inflammation was induced. Thereby, ROS are the key signaling molecules to be produced during inflammatory processes. Subsequent DNA damage activates the immune system and the production of cytokines [14,15]. IL-8 is a protein that is barely secreted from non-induced cells, but its production increases rapidly by stimuli such as pro-inflammatory cytokines and bacterial products by more than 100-fold [13].

For LPS, it was found that the inflammatory potential on TR146 cells was negligible. LPS is known to bind to TLR located on epithelial cells, more specifically to TLR-4, inducing IL-8 release. However, TR146 cells mainly express TLR-1, 3, and 6 and TLR-4 only to a small extent [46,47]. Hence, the missing TLR-4 prevents interaction of LPS with the buccal epithelial cells and impedes the stimulation of inflammation. IL-1α plays a crucial role in the signaling of pathologic conditions in the oral cavity. Sorenson et al. investigated the role of IL-1α in activating the antimicrobial protein complex calprotectin to activate the innate protective barrier in order to counteract bacterial or fungal invasion. They verified the potential of IL-1α to trigger protective signaling pathways [48]. By activating the innate immune system, further pro-inflammatory cytokines were released, which essentially led to inflammation. In our study, it was shown that IL-1α alone was not potent enough to trigger inflammation in TR146 cells. Although the highest concentration led to a significant decrease in cell viability and membrane integrity, resulting in a loss of cell functions, no increase in ROS or IL-8 release was observed. This suggests that IL-1α triggers an apoptotic event in TR146 cells but needs immune cells to activate the innate immune response and/or additional pro-inflammatory mediators to induce IL-8 release. Besides IL-1α, also IL-1ß and TNF-α play an essential role in inflammatory processes. More specifically, both are involved in the development of diseases such as periodontal disease and oral mucositis [9,10]. For example, oral mucositis is a side effect of radiation therapy for the treatment of head/neck-cancer. Radiation therapy causes DNA and non-DNA damage and the generation of ROS in the basal epithelium and in the submucosa [49]. This results in the activation of transcription factors and others, which induce upregulation of genes and consequently, the production of pro-inflammatory cytokines including TNF-α, IL-1ß, and IL-6. As a consequence, thinning of the epithelium occurs because of reduced cell proliferation [50]. Our results showed that TNF-α did not influence the cell viability but to a small extent the membrane integrity. This is due to the fact that TNF-α induced a restructuring of the actin-myosin filaments in TR146 cells, resulting in swelling and leakage of the cell membrane. In addition, ROS and IL-8 levels increased, which coincides with the literature as TNF-α increases IL-8 release more than 100 times in response to cytokine release, bacterial products and cellular stress [13,51].

Moreover, IL-1ß and TNF-α induced cell morphological changes. The cell membrane consists of different proteins and lipids, particularly phospholipids, which determine the charge of the membrane by their arrangement. Thereby, the neutral phospholipids such as phosphatidylcholine and sphingomyelin are located in the outer leaflet. The inner leaflet comprises higher amounts of negatively charged PS and phosphatidylethanolamine [52]. Due to the asymmetric distribution of these phospholipids, intact cell membranes exhibit a neutral net surface charge. Cell damage, oxidative stress, cytokine exposure, and cellular changes such as increased influx of calcium ions into the cytoplasm and ATP depletion can lead to the translocation of PS to the outer leaflet of the cell membrane, which has been previously reported for a variety of epithelial cancer cell lines (e.g., melanoma, breast cancer, kidney, brain, prostate, etc.) [53,54,55]. This suggests that lipid translocation switches the surface charge of the cell membrane, more precisely, the once neutral charge of the cell membrane changes to a negative charge. Furthermore, translocation of PS is an indicator of early apoptosis [56,57]. Our data showed that IL-1ß and TNF-α induced a lipid re-structuring in TR146 cells and thus, changed the net surface charge towards negative. However, the tested concentrations of IL-1ß and TNF-α (i.e., 400 ng/mL and 300 ng/mL) did not show any signs of cell death. Only higher concentrations (i.e., >800 ng/mL for IL-1ß and >400 ng/mL for TNF-α) lead to a decrease in the cell viability and/or increase in ROS production. For all tested cytokines, PS-exposing clusters as well as small PS-exposing blebs were detected in the outer leaflet of the cell membrane. They fulfill important functions including intercellular communication especially during inflammation, cell division, cell movement, protection of the cell membrane during cell injury (i.e., damage-control mechanism), and apoptosis [58,59]. Previously, similar blebs and morphological alterations were reported for TR146 cells after incubation with dental casting alloys. Together with increased TNF-α expression leading to apoptotic events, all these factors indicated the initiation of inflammation. [60]. Apart from the cell membrane, re-organization of the cytoskeleton is known to occur during pathological disorders that involve inflammation [41,61,62]. This is important to investigate, as the cytoskeleton has important functions such as morphogenesis, migration, cytokinesis, endocytosis, and phagocytosis. In TR146 cells, it was found that the cytoskeleton was reorganized, and stress fibers were formed. This coincides with the literature as small amounts of TNF-α induce stress fiber formation and cytoskeletal rearrangement in rat fibroblasts. Thereby, approximately 60–70% of TNF-α-induced cytoskeletal reorganization is mediated by ceramide signaling, which in turn activates the sphingosine kinase and leads to the formation of stress fibers [63].

In the oral mucosa, epithelial cells are connected via desmosomes, which are responsible for the elasticity and mechanical stability of the tissue [61,64]. These calcium-dependent intercellular junctions form a supracellular scaffold by anchoring intermediate filaments to the plasma membrane [62]. During inflammatory events, desmosomes are down-regulated to enable cell migration and healing [65,66]. Thereby, it has been found that Desmoglein 3, the calcium-binding transmembrane glycoprotein component of desmosomes, is mainly present on the cells of the oral mucosa [67]. The results of the cell–cell contact studies demonstrated that the monoclonal anti-Desmoglein 3 antibody was not able to bind to the autoantigen Desmoglein 3 of intact TR146 cells. By contrast, after treatment with IL-1ß and TNF-α, binding of primary and secondary antibodies was detected by an increase in the red fluorescence intensity. Thus, both cytokines led to a loss of cell–cell contacts and consequently to the exposure of Desmoglein 3. Clinically, a loss of cell–cell contacts is characterized by blisters and erosions of the oral mucosa. It is assumed that blisters are produced by anti-Desmoglein 3 antibodies through steric hindrance, Desmoglein 3 internalization into endosomes with the goal of degradation, alterations in molecular integrity, or changed cell signaling pathway [64,68]. Moreover, both cytokines restricted the proliferation of TR146 cells. As a result, an increased permeability of the oral mucosa can be expected. These findings correlate well with studies performed in the intestine. Elevated TNF-α levels led to cell shedding at the villus tips and altered the cellular junctions, resulting in an increased intestinal permeability [69,70,71]. Besides TNF-α, it was found that IL-1ß was also overexpressed in inflamed intestinal tissue and contributes to damage [72]. In addition, it was found that both cytokines reduced proliferation of intestinal epithelial cells [73,74].

In order to investigate how these changes influence interactions with drug delivery systems, we tested lipid nanocarriers, so-called NLC. Initially, comprehensive studies were performed on untreated TR 146 cells to exclude possible influences of lipid particles on changes in cell morphology, influence on membrane integrity, and apoptosis. Interestingly, the ROS production of untreated cells was increased. According to the literature, it is known that nanoparticles can interact with mitochondria, resulting in mitochondrial damage and consequently ROS production [75]. Although mitochondrial activity was not affected by NLC, ROS production increased after exposure but was lower than the lowest positive control. This suggests that the particles induced a brief stress response in the cells, but no significant IL-8 release. In addition, the cell morphology of TR146 cells was not affected (i.e., intact cell–cell contacts, no stress fibers, and no PS translocation), indicating that NLC do not induce adverse effects.

In general, the uptake mechanism of lipid particles depends on the cell type, and the toxicity profile is determined by the lipid composition [76]. Another important parameter that needs to be considered in the mouth is the particle size. The epithelial cell surface shows ridge-like folds, referred to as microplicae. These microplicae enhance the effect of size-dependent uptake based on thermodynamic driving forces. The diameters of buccal microplicae range from 200 to 500 nm. Once particles have the appropriate size to deposit there, i.e., larger than 200 nm, interparticle electrostatic repulsion is reduced and cellular uptake is enhanced compared to smaller particle sizes [25]. Preliminary studies performed by our group showed that 280 nm-sized NLC based on palmitic acid and oleic acid actively interacted with healthy oral excised tissue and transported the drug into the first third of the epithelium [33]. In cytokine-stimulated TR146 cells, it was found that although desmosome opening was induced, the uptake mechanism of NLC remained the same and occurred exclusively via caveolin-mediated endocytosis. Because of the change in cell membrane charge from neutral to negative, repulsive interactions between the hydrophobic negative NLC and the cell membrane prevented particle adhesion to the membrane and facilitated hydrophobic interactions with lipophilic proteins in the cell membrane surface [25,77]. In addition, the observed decrease in membrane integrity further promoted the increased NLC uptake. To the authors´ knowledge, there are no data available on the uptake mechanisms of lipid particles into the buccal epithelium to confirm these assumptions. However, the results correlate with the absorption of NLC in the small intestine, which is also suggested to occur via caveolin-mediated endocytosis [78,79,80]. Moreover, Bae and coworkers showed that a mixture of IL-1ß and TNF-α significantly increased the expression of caveolin-1 in pancreatic INS-1 cells [81]. Hence, it seems likely that overexpression also occurs in TR146 cells, which leads to an increased number of caveolae and finally enhances the uptake of NLC under inflammatory conditions.

## 5. Conclusions

Inflammatory diseases of the mouth, such as stomatitis and oral mucositis, are chronic conditions pathologically characterized by inflammation and epithelial lesions. Treatment, particularly with locally applied therapeutics, is currently limited, although side effects can be significantly reduced, and application is facilitated for dry mouth and dysphagia. Using human epithelial TR146 cells, it was found that cytokines, in particular IL-1ß and TNF-α, induced an inflammatory event in TR146 cells. This resulted in cell membrane restructuring, formation of stress fibers, loss of cell–cell connections, change in the membrane charge, and decreased cell proliferation, leading to an altered barrier integrity. The changes did not affect the cellular transport mechanism, i.e., caveolin-mediated endocytosis per se, but increased the uptake capacity of negatively charged NLC due to a decrease in membrane integrity. An optimum particle size of approximately 280 nm further promoted hydrophobic forces, which were more pronounced over shorter distances than the electrostatic repulsive forces between the negatively charged membrane and the negative particles. In addition, the use of cationic lipids could reduce these electrostatic repulsive forces to a minimum and thus further enhance the interaction with the cell membrane. Moreover, overexpression of caveolin-1 contributed to an increased NLC uptake in TR146 cells, which will be further investigated. In addition, preliminary studies excluded that the NLC particles exert negative effects on non-treated cells. It must be noted here that the data are based on studies of a simple cell system. To perform even more complex studies and thus build a deeper understanding, a co-culture model is needed to represent the anatomy and physiology of the oral mucosa in the diseased state in more detail.

## Supplementary Materials

The following are available online at https://www.mdpi.com/article/10.3390/nano11051330/s1, Supplementary Figure S1: Fluorescence microscopic images (overlay of bright field and fluorescence channels) of TR146 cells treated with 800 ng/mL IL-1ß (A) and 400 ng/mL TNF-α (B). Arrows indicate specific binding of Annexin V-Alexa Fluor 488 (green) to PS on the outside of the cells. Stars show representative PI stained necrotic cells (red). Supplementary Figure S2: Fluorescence microscopic images of untreated TR146 cells (A) and TR146 cells treated with ETDA (B), 400 ng/mL IL-1ß (C) and 300 ng/mL TNF-α (D). Desmosomes (red) were stained with anti-Desmoglein 3 antibody and binding visualized with a secondary antibody conjugated to Alexa Fluor 568.
