# Supplementary material for: Cytokine-Mediated Inflammation in the Oral Cavity and Its Effect on Lipid Nanocarriers
Source: Nanomaterials (Basel). 2021 May 18;11(5):1330. doi: 10.3390/nano11051330 (PMC8157841; doi:10.3390/nano11051330)
Supplement: Supplementary file 1 [file nanomaterials-11-01330-s001.zip › nanomaterials-1213091-supplementary.pdf]

Supplementary Material

Cytokine-Mediated Inflammation in the Oral Cavity and Its Effect on Lipid Nanocarriers

Carolin Tetyczka [1], Sonja Hartl [1], Ramona Jeitler [1], Markus Absenger-Novak [2], Claudia Meindl [2], Eleonore Fröhlich [2], Sabrina Riedl [3], Dagmar Zweytick [3] and Eva Roblegg [1,*]

[1] Institute of Pharmaceutical Sciences, Department of Pharmaceutical Technology and Biopharmacy, University of Graz, 8010 Graz, Austria; carolin.tetyczka@uni-graz.at (C.T.); sonja.hartl@uni-graz.at (S.H.); ramona.jeitler@uni-graz.at (R.J.)

[2] Center for Medical Research, Medical University of Graz, 8010 Graz, Austria; markus.absenger@medunigraz.at (M.A.-N.); claudia.meindl@medunigraz.at (C.M.); eleonore.froehlich@medunigraz.at (E.F.)

[3] Institute of Molecular Biosciences, Biophysics Division, University of Graz, 8010 Graz, Austria; sabrina.riedl@uni-graz.at (S.R.); dagmar.zweytick@uni-graz.at (D.Z.)

* Correspondence: eva.roblegg@uni-graz.at; Tel.: +43-(0)-316-380-8888

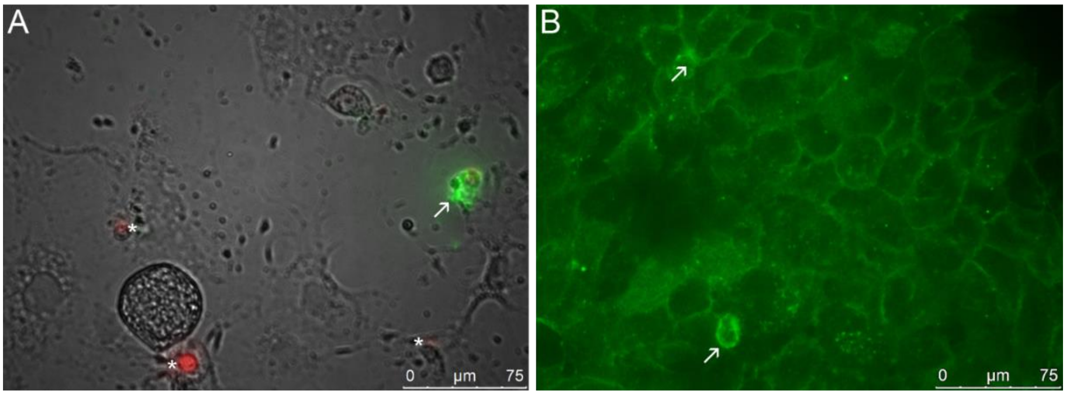

Figure S1. Fluorescence microscopic images (overlay of bright field and fluorescence channels) of TR146 cells treated with 800 ng/mL IL-1ß (**A**) and 400 ng/mL TNF-α (**B**). Arrows indicate specific binding of Annexin V-Alexa Fluor 488 (green) to PS on the outside of the cells. Stars show representative PI stained necrotic cells (red).

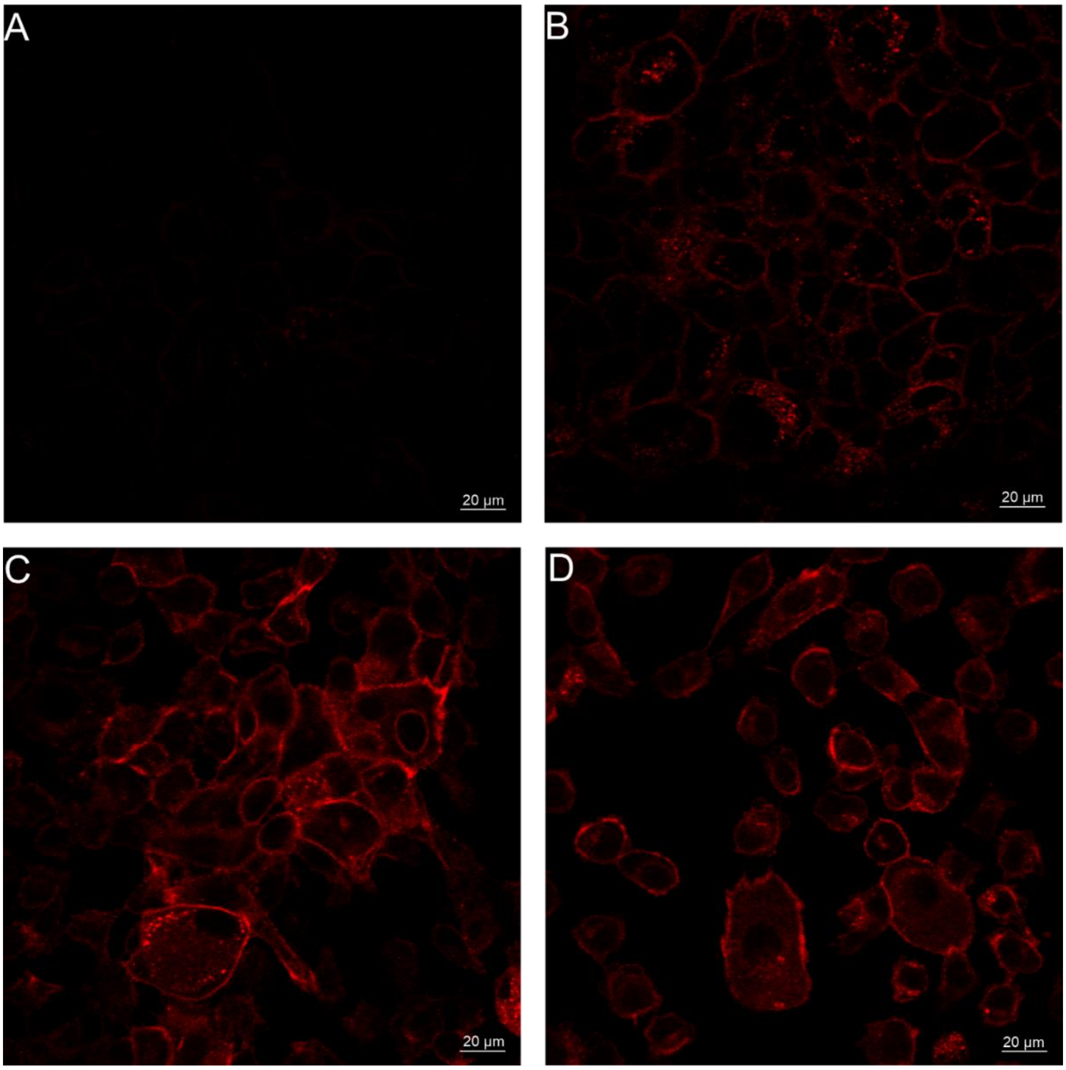

Figure S2. Fluorescence microscopic images of untreated TR146 cells (**A**) and TR146 cells treated with ETDA (**B**), 400 ng/mL IL-1ß (**C**) and 300 ng/mL TNF-α (**D**). Desmosomes (red) were stained with anti-Desmoglein 3 antibody and binding visualized with a secondary antibody conjugated to Alexa Fluor 568.
